# The Development of Sensor Applications in the Sectors of Energy and Environment in Italy, 1976–2015

**DOI:** 10.3390/s17040793

**Published:** 2017-04-07

**Authors:** Girolamo Di Francia

**Affiliations:** Italian National Agency for New Technologies, Energy and Sustainable Economic Development (ENEA), P.le E. Fermi 1, Napoli 80055, Italy; girolamo.difrancia@enea.it; Tel.: +39-81-772-3277

**Keywords:** sensor, energy, environment, WSN, e-nose, smart-meter, e-tongue

## Abstract

Although sensor technologies have been developing quite similarly all over the world, the investigation of their applications has been more affected by the specific industrial and economic characteristics of each country. This paper aims to investigate the development of applications based on sensor devices in the sectors of energy and the environment, in Italy, throughout the last forty years (1976–2015), examining the most relevant papers published by Italian R & D groups working in this field. Italy depends on foreign imports for more than 80% of its primary energy needs, and this has directed the research effort on the development of sensor applications both to improve load shaping and consumers’ awareness and to develop specific equipment to maximize renewable energy production. Similarly, for the environment sector, there are increasing efforts to develop solutions to support a more and more capillary control of the environment itself using a cooperative approach. In both the sectors it seems that the solutions proposed can help to relieve the structural problems that Italy suffers and that the scientific and technical results obtained so far also have significant international relevance.

## 1. Introduction

This review is meant to be one of the papers included in the special issue that Sensors is dedicating to “State-of-the-Art Sensors Technologies in Italy” and, in particular, its aim is to provide a snapshot of the research activities that have been running in this country in the last forty years to develop solid-state sensor applications in the sectors of energy and the environment. It does not make any claims of completeness and, in particular, it will not discuss the sensor technologies themselves since other papers included in this special issue will deal with these topics in more detail.

For the sake of clarity, it is worth noting that the papers selected for this review have been chosen, among those published in the time period considered, according to three major criteria:

(1) Novelty of the research topic discussed in the selected paper, in the Italian context;
(2) Compliance of the research results reported in the selected paper with the best results published, contemporarily, at the international level;
(3) Relevance of the specific theme discussed by the selected paper with respect to the Italian context.

**Figure 1:**
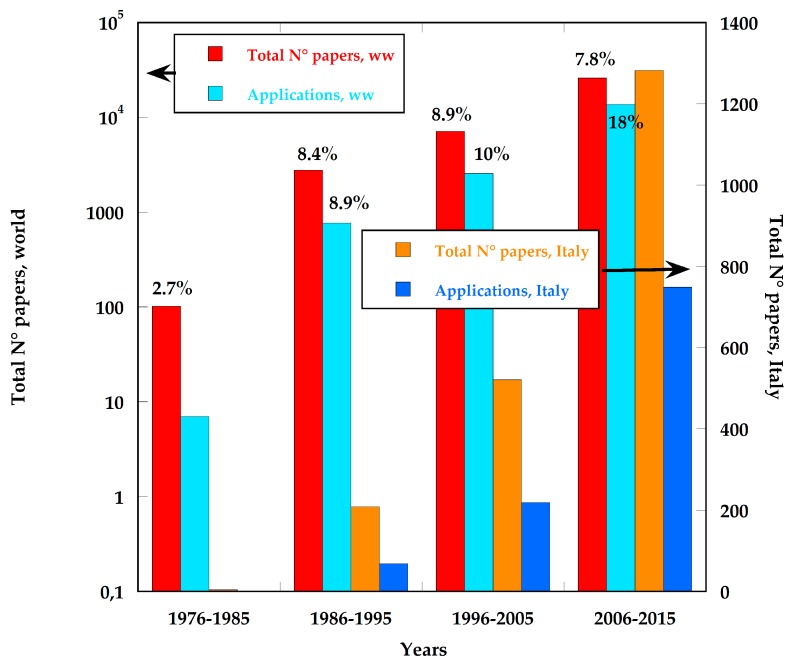
Scientific and technical papers concerning the development of sensor devices and of related applications, published in the period 1976–2015, both worldwide and in Italy. At the top of the columns, the ratio (%) of citations of Italian papers to the total (ww) number of citations (self-citations excluded) is reported, for each category considered.

By the seventies, the research activities in the field of solid-state sensors had seen, worldwide, a progressive but impressive shift from studies related to the investigation of single devices, optimized for a definite target (i.e., a specific air pollutant), to the first studies, during the nineties, devoted to investigating topics more and more related to application scenarios, such as arrays of sensors, e-noses, smart energy meters, etc., with the corresponding development of technologies for sensor integration and communication and of computerized numerical methods to handle larger collections of data. In Figure 1 the total number of papers concerning sensor development, as reported by the major specialized scientific and technical journals of the sector, is shown for the four decades in question, both worldwide (ww) and in Italy; for comparison, it also shows the number of papers more related to the development of specific applications, worldwide and in Italy [1].

Both worldwide and in Italy, the impressive growth of the sector as a whole is noteworthy, as seen by the steady increase in the number of scientific papers published and, moreover, by the even greater increase of more application-oriented works, accounting in the last decade to about 52% of the total number of papers (36% in the previous decade). Of course, this rapidly evolving “change of paradigm” has only been made possible as a result of the explosive growth of the ICT technologies that have made microprocessors and electronic devices available to economically handle very large amounts of data, opening the way for the development of innovative applications. As a result, also in the sectors of energy and the environment, small equipment devoted to the monitoring of energy consumption or to the analysis of gaseous and liquid matrixes, such as smart energy meters, e-noses and e-tongues, has been developed and specialized for different applications [2,3,4]. The new millennium has finally seen the explosion of sensor research related to the more and more pervasive applications of wireless sensor networks (WSN), which very often rely on the Internet to share and even to produce data. Italy, as it will be shown below, has also followed this trend, although with some specific peculiarities typical of the country. It is worth noting that in Italy the growth of more application-oriented research activities has been even more marked than in other countries: in the last decade, almost 60% of all the published research has been oriented towards application scenarios (as opposed to 42% in the previous decade). It is even more interesting to see that the results obtained have also received great interest in the scientific community, since almost 18% of all the works cited in the last decade have been produced by Italian groups.

## 2. Energy

Energy and environment are strictly related, the former having been claimed as responsible for most of the emissions of greenhouse gases and air pollutants in Western societies [5]. It is therefore only for simplicity that hereafter the two topics will be discussed separately. The energy sector is intended here as the ensemble of activities devoted to the production and usage of the energy required by all the other economic sectors (transport, domestic, industry, agriculture, services, etc.).

Throughout most of the last century, European countries have strongly differed in terms of energy dependence and, as a consequence, in terms of their energy policies. It was only when the Common European Market was envisaged, in the late eighties, that the member states finally agreed on the adoption of a common strategy both in terms of energy resource exploitation and in terms of environment protection, trying to close the gap with other, more advanced countries such as the US where, for instance, the first Air Pollution Control Act dates back to 1955. As far as Italy is concerned, apart from a few attempts during the sixties and the seventies, the country’s energy policy has been characterized by a marked incapability of long-term planning [6] and, as a consequence, up to the end of the nineties the research effort to develop sensor applications in the energy sector turned out to be very limited, mostly due to ENEL, the only Italian operator active in the production and distribution of electric energy, with research mainly focused on the optimization of the combustion process and the study of electric cable reliability [7,8].

**Figure 2:**
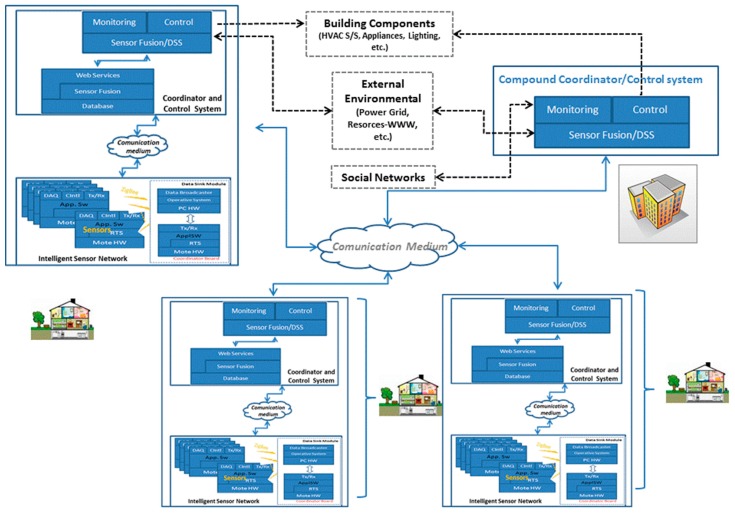
The architecture of the proposed pervasive energy usage monitoring system discussed in [14]. It has been shown that pervasive and continuous power monitoring in households allows energy savings between 10% and 15% to be achieved.

As mentioned above, the situation only changed during the last 20 years when, following the EU strategies, Italy became fully aware of: (1) the environmental problem related to energy production and use and (2) the necessity of adopting policies capable of significantly reducing the at least 40% loss in energy use by putting into practice the concepts of efficient energy exploitation. Strategies of energy production optimization and load shaping were at first proposed, suggesting the use of systems designed to improve data production on the consumers’ side [9,10]. But the concept of energy efficiency became really popular only when, also in this country, systems and techniques for distributed process control and optimization turned into practical applications, with the first examples being light management in buildings [11] and with the design of WSNs devoted to the control of industrial processes [12]. With the gradual decrease in the cost of WSN solutions, this concept began to be implemented in civil contexts with the basic raw data required to carry out control and optimization strategies, provided not only by light sensors but more and more often also by indoor air quality (IAQ) detectors, real time energy meters and later on, with the growth of Web based services, by means of systems to monitor human activity in the smart home and office [13,14,15,16,17]. Figure 2, for instance, shows the architecture proposed in [14] which consists of three main components: a base station, two types of smart energy meters and web-based GUIs. In order to monitor the power usage of any type of electric appliance, two types of energy meters have been designed and developed: a power outlet adapter and a clamp based smart energy meter. The architecture is basically built up around a universal converter for true RMS current measurement and a TelosB Mote TPR2420. Embedded software components, coded in NesC, allow local data acquisition and processing while the runtime support of the open-source operating system, TinyOS, provides basic functionalities such as network formation, packets routing and management of the mesh topology.

The successful application of this approach to building energy management was then rapidly extended to data centers, which now account for about 2% of electric energy use in Western countries. Global energy management solutions also began to be proposed as retail solutions relying on the collection and processing of sensor data on web services and, therefore, using the Internet as an integrated solution [18]. This rapidly converged towards the smart city concept, even if the first practical applications were again found in buildings [19,20,21]. It is worth noting that in Italy, where most of the buildings are quite dated, innovative retrofitting solutions have been specifically investigated [22]. Recent works have for instance developed solutions where light and motion sensor nodes, implementing ZigBee communication stack generating pulse width modulation signals, control the LED illumination equipment that are becoming more and more popular in our homes [23]. Such a minimally invasive approach has been also further investigated to measure energy consumption in a WSN of common amperometric sensors [24]. A further recent research investigation field typical of Italy is the connection of the indoor WSN reconstructed reality to external ambient conditions such as the weather conditions. This approach tries to take into account the site-dependent variation of the external conditions in a learning-by-example strategy to improve indoor thermal comfort [25].

**Figure 3:**
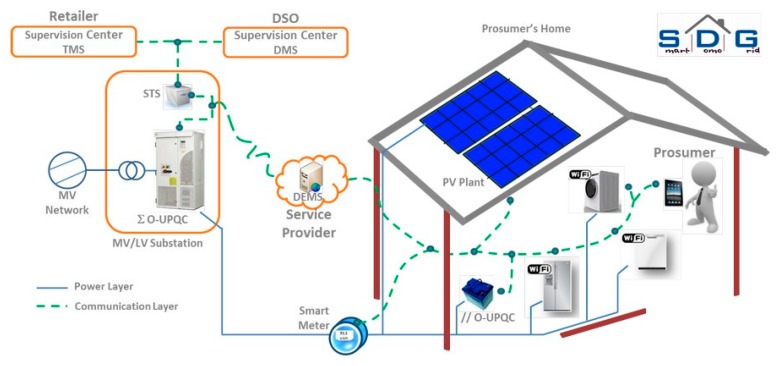
Structure of the test bed used in the Italian project Smart Domo Grid (http://www.serviziarete.it/progetto-smart-domo-grid-a2a/, last accessed January 2017) to monitor in real time the power exchanged between a customer and the electric grid in terms of active and reactive power, line frequency, voltage dips, and harmonic distortion.

With respect to energy production in Italy, the last 20 years have been a transition period, mainly characterized by an energy policy that, following the EU strategy, has paid increasing attention to renewable energies (wind, photovoltaic, etc.). It is worth noting in this respect that Italy leads, at present, among EU countries in terms of the amount of electric energy produced by photovoltaic (PV), with 8% of its annual production from this specific energy source. Sensors have played a major role in this evolution scenario, both in terms of production process optimization [26,27,28,29] and, more recently, in the development of smart grids [30,31]. Due to the relevance that PV has in our country, research has recently been greatly focused on this field, and smart systems designed to continuously monitor a PV plant, based on a WSN with nodes associated to each PV module, have been proposed to measure the plant performance, optimizing its operation and preventing failures [32]. It is worth stressing that this type of system can be installed in existing plants in a retrofitting approach. Similar attention has been paid to the problem of sensor deployment to maximize irradiance in a large PV field. This problem is crucial to optimize energy production and its commercialization so that innovative interpolation algorithms have been proposed and tested to be fully operative with a finite, minimum number of detectors [33]. In this context, and since renewable energies strongly depend on the weather conditions, sensors for weather forecasting and techniques for energy production optimization and transmission that take into account the great number of distributed generators characterizing this type of energy source are being investigated more and more. Here again the first and more widely explored scenario is a building with a focus on the net zero energy scenario (NZEB) where sensors and microcontrollers are distributed in correspondence with each source/load in order to give a very accurate description of real time energy production/use [34]. The research field focused on monitoring the low voltage electric grid status using the new generation of smart meters that are actually being installed in Italian homes is also quite interesting [35]. Here the problem investigated is related to the assessment of the quality of the electric energy that is being distributed, taking into account the large pervasiveness of small/medium renewable energy plants that characterize the Italian case. Figure 3 shows the structure of a test bed used in the city of Brescia to investigate the possibility of using the new meters to monitor the electric energy distribution grid.

## 3. Environment

As far as the research for innovative environmental sensor applications is concerned, a great step in this field came worldwide from the US in 1967 with the revision of the Air Quality Act, which strongly supported the development of new electronic instrumentation for air quality control [36]. In Italy however the first laws and regulations in this sector were only approved 21 years later (law DPR n 203, 1988) resulting in a slower development of the related research activities in spite of the fact that a few papers had been already published on these topics at the beginning of the seventies [37,38]. Several Italian groups began then to investigate these fields [39,40,41,42] and it was mainly as a result of that pioneering effort that the national association of the research groups involved in the field of sensors and microsystems, AISEM, could be founded in 1995.

**Figure 4:**
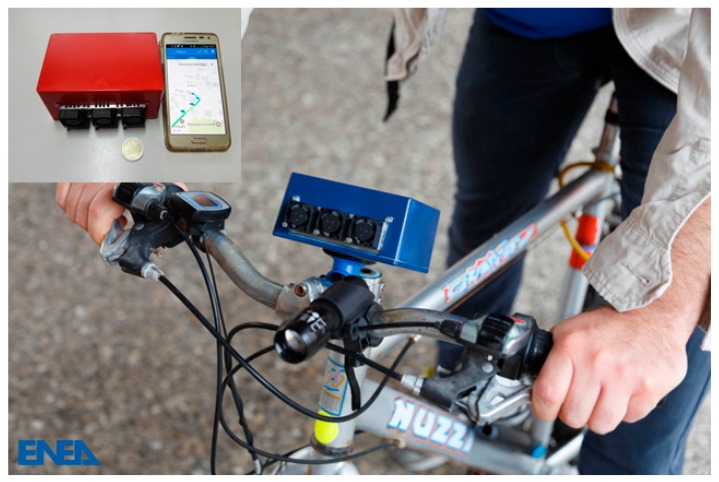
The Monica ^TM^ system is a solution for cooperative urban pollution monitoring [53]. It relies on an STM32 microprocessor and is equipped with an NO_2_, CO, RH, T and O_3_ sensors. Data can be sent to a smartphone and shared with other users to construct a real-time pollution map of the city.

As far as air pollution is concerned, there was initially great interest in the main gaseous pollutants SO_2_, CO and NO_2_ [43,44,45], and to then investigate the detection of benzene [46], ethanol and methanol [47] or, even using optical fibers, other VOCs such as xylene and toluene [48]. Sensor arrays for the detection and discrimination of multiple pollutants in the air were also investigated [49] taking into account the experience in the realization of the first Italian electronic noses [50,51]. More recently, portable monitoring systems, as it is for instance shown in Figure 4, have been developed and integrated taking advantage of strategies of cooperative urban monitoring, eventually with the support of smartphones [52,53].

It is interesting to observe that this development path has been accompanied by an increasing interest in the definition of calibration procedures both for sensor devices [54] and for array of sensors [55,56,57] and in the definition of suitable air quality synthetic indexes [58]. Innovative approaches to handling problems such as rapid transients in pollutants concentrations in urban environment have been discussed in terms of dynamic neural networks both theoretically and with field tests, taking into account the slow dynamics of solid-state sensors and their intrinsic cross interference problems [59]. Such an approach seems particularly interesting in the frame of mobile cooperative monitoring issues. Along with this research, IAQ has found an increasing interest in the scientific community since the beginning of the nineties [60] for its possible applications in public buildings such as museums [61] and also for odor annoyance assessment [62], giving rise to an intense development of specialized electronic noses [63,64,65] or of flexible architectures capable of tuning the e-nose sampling recipe to analyze different situations and to generate odor maps [66].

**Figure 5:**
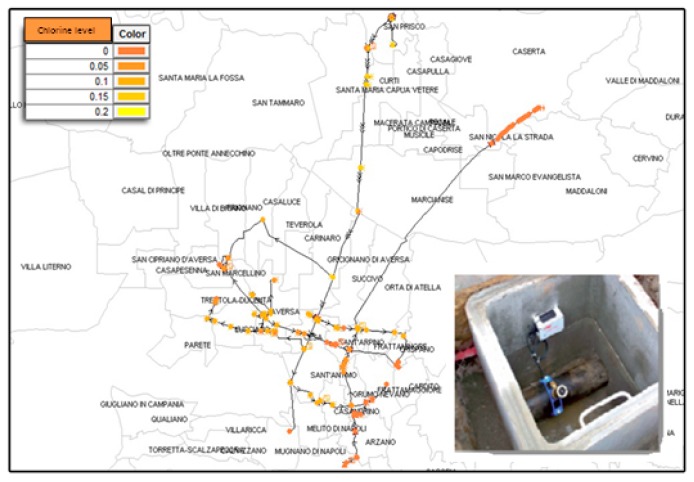
The prototype of a system for smart water metering (details in [77]). The system is actually under test in the Santa Sofia aqueduct, near Napoli.

As far as liquid environments and in particular water are concerned, since the late nineties several prototypes of electronic tongues have been developed to assess water quality [67]. In comparison with air, water is characterized by less assessed scenarios, so several technologies are still under investigation. Sensors based on optical fibers have been tested for pH, nitrates, and ammonia [68,69,70], and enzyme-based biosensors have been tested for sulfites and phenols, monitoring both waste and marine or river waters [71,72]. More recently, also in this context and, in particular, to monitor and control water distribution networks, the concept of WSNs has been considered [73] for marine environments [74,75] and to develop a sort of smart meter for water distribution networks [76]. It is worth noting that this scenario is becoming more and more relevant for Italy and that original approaches based on innovative sensors for liquid level and quality, WSN data extraction and modeling techniques even using suitable hydraulic software and GIS tools have been proposed for the first time to monitor drinking and waste water quality [73,77]. In Figure 5 an example of one of such approaches is, for instance, shown.Portable equipment based on sensor arrays has been finally developed [78]. Arrays of porphyrin-based sensors differently functionalized, illuminated under an LED light so to produce different color images detectable even by a smart phone camera and useful to reveal the presence of drugs and pesticide, have been for instance proposed [79] and, as far as marine environment is concerned, a specialized e-nose capable to detect hydrocarbons in the sea water using an array of PID detectors has been also reported [75].

It is finally worth noting that, more recently, the soil matrix has also begun to be investigated by means of solid-state sensors. In this case, however, most of the research activities so far published are still concentrated on the determination of single parameters such as moisture, carbon content [80,81] or soil resistivity [82].

## 4. Discussion

The sectors of energy and the environment are very critical for the Italian economy. In the former case, Italy has a chronic, strong dependence on foreign imports for its primary energy needs, while in the latter case the urgency is due to the lack of attention from national government policies to the problems arising from air, water and soil pollution.

This paper has shown how the scientific research in the field of sensors has been affected by these emergences and, in turn, how this situation has driven research groups’ activities to develop innovative specific solutions. In both cases it has been shown that the need to fulfill the requirements set by European directives has greatly contributed to defining the framework for the development of solutions tailored to the country’s needs. As far as energy is concerned, sensor applications for improving energy efficiency have been developed, in particular for buildings, and solutions to optimize the energy production of renewable sources and their integration in the grid have been proposed. Research dedicated to the environment is, on the other hand, increasingly focused on the development of solutions for widespread and distributed cooperative monitoring of air, water and soil, and also taking advantage of web-based services. It is finally worth noting that Italian researchers are increasing their scientific relevance in the development of sensor-based applications, as can be observed by the marked increase in the number of Italian papers cited in the last decade, although at the price of less impact on basic research activities.
